# Dr. Edward Maynard

**Published:** 1891-06

**Authors:** H. W. S. Cleveland


DR. EDWARD MAYNARD.—The announcement of the death at Washington, D. C., on Monday, May 4, 1891, of Dr. Edward Maynard, at the ripe age of more than seventy-seven years, affords a striking illustration of how speedily a man whose name was widely known and honored in his day of active service may drop out of sight and be almost forgotten in the turmoil and seething fermentation of succeeding years, when a new race has come to the front and is absorbed in present issues, while the deeds of yesterday are only "remembered as a tale that's told."

Had Dr. Maynard's death occurred twenty-five or thirty years ago there would have been no need of telling the world who or what he was. Few of his contemporaries are left to-day. To them the mention of his decease will awake many reminiscences of long past years, but to the great mass of readers it will be unknown.

He was a man of rare qualities and of rare acquirements, and apart from the elements of character which commanded the respect of all who knew him, and the warm affection of the wide circle of his friends—the world at large is indebted to him for discoveries the value and importance of which can never be justly estimated, and ought to be gratefully remembered.

He was born in Madison, N. Y., April 26, 1813, entered the Military Academy at West Point in 1831, but owing to delicate health was forced to resign the same year. He then applied himself to the studies of civil engineering, architecture, anatomy and drawing, with an earnestness which was a prominent characteristic, and which could never be satisfied with smattering acquirements or with half-way work. He finally adopted the profession of dentistry, and established himself in Washington in 1835.

His discoveries in dental surgery have been of such importance and his skill as an operator was so remarkable, that it may be safely said that he has had no superior in his profession, and the honors bestowed upon him in recognition of it in this country and in Europe are sufficient proof of the truth of the assertion.

Many of the most important improvements in the instruments of dentistry were of his invention. He was the first to successfully practice (in 1838) the thorough filling with gold foil of the nerve cavity, including the nerve canals in molar and bicuspid teeth. He introduced this operation in Europe in 1845, and at St. Petersburg. The Emperor's physician, Dr. Arndt, having witnessed it, Dr. Maynard was immediately employed as the court dentist. The Emperor (Nicholas I.) offered to give him a title with the rank of major if he would remain in Russia ten years and teach and practice his profession while attached to the court. This he declined, but received from the Emperor in addition to the sum paid for his services a magnificient diamond ring.

In 1857 he accepted the chair of Theory and Practice in the Baltimore College of Dental Surgery (the first dental college ever established), and held the like position in the Faculty of the Dental Department of the National University at Washington. He received honorary degrees from leading associations in Europe and America, and for a long series of years his practice was among the highest classes in Washington, including several Presidents and a great number of Cabinet officers, Senators, Representatives, officers of the Army and Navy, Foreign Ministers, and others who demand such operations as necessarily exclude all machine work.

His winning and always gentlemanly and courteous manner, his rare intelligence and the wide scope of his information, served to secure the warm personal friendship of all whom he chose to admit to such intimacy, and the large and varied circle of acquaintance he thus made with leading men of all parties, sects and opinions, gave a rare zest to his conversation and made him a most interesting companion.

But the honors and friendships he acquired through his professional reputation and practice were equalled if not surpassed by those resulting from the exercise of his inventive talents in another and entirely different field. Thousands of men in every quarter of the globe who were ignorant of the fact that he was a professional dentist are familiar with his name in connection with the Maynard rifle, which was the first breechloader that proved itself equal in its performance to the best muzzleloading rifles, and may be truly said to have served as the model which has revolutionized the arms of the civilized world. This is neither the time nor the place for detailed statements of its peculiar features, but there are circumstances connected with its history, that in justice to the memory of the inventor should be clearly stated and put on public record.

The invention was patented several years before the war, and therefore before the demand for improved weapons had incited inventors everywhere to the study of the subject. There had been many previous attempts to construct breechloading guns, but the results had been so utterly inadequate to the wants of military service and so far below the required merits of the sportsman that they served rather to illustrate the difficulty of the problem than to aid in its solution.

The perfect simplicity and entire efficiency and safety of the system of levers by which in the Maynard rifle necessary movements of the barrel are effected can only be compared to the anatomical system by which the greatest possible ease of motion and resistance to pressure are secured in the animal structure. Yet the ingenuity here displayed was really of secondary importance compared to that of the ammunition and the method of its preparation, which involved the principle by which alone it has thus far been found possible to secure in breechloaders the same degree of precision and force that is attained by muzzleloaders.

Dr. Maynard was the first inventor of a metallic centerfire cartridge, and the instrument by which it was loaded insured the perfectly true delivery of the bullet into the barrel of the rifle, and the most exacting tests to which it was subjected served to prove that it had no superior in all essential points, while in facility of manipulation it so far excelled all others that it was at once obvious that a revolution in firearms was at hand. Innumerable efforts at improvements have since been made and are still making, many changes of models of guns have been the result, in adapting them to the necessities of modern military service, or increasing their efficiency by various ingenious devices, but in all of them the scientific principles by which

Dr. Maynard first secured the prime essentials of precision and force are still adhered to and have never been improved upon. Various changes of detail in the Maynard rifle have from time to time been introduced, but in all its essential features it is the same as the original weapon and still holds its high place in the estimation of leading sportsmen and riflemen throughout the country.

Dr. Maynard has himself made many additional inventions in connection with firearms of very great value, as for instance a register which may be attached to any repeating rifle, which indicates at all times the number of cartridges that are in the magazine. He has also made a double-barreled rifle which completely overcomes the previously inevitable defect of unequal accuracy owing to the deflection of one barrel by the heating of the other. Instead of soldering the barrels together he simply clamped them by a most ingenious device, which while perfectly strong and firm, allowed the expansion of either barrel without affecting the other. Another advantage was that if desired a shot barrel might in a moment be substituted for one of the rifle barrels, or a pair of shot barrels in place of the rifles. A very beautiful model of such a gun was made and its efficiency proved by careful tests, and so high an authority as Quartermaster-General Meigs said of it that if introduced it would drive all other sporting guns from the market. But it has never been manufactured for sale, and the model alluded to is the only one in existence.

The Kings of Sweden and Prussia recognized the great value of Dr. Maynard's inventions. The former by giving him the Great Medal of Merit of Sweden, an honor rarely conferred, and the latter by decorating him with the Cross of the Red Eagle.

The very imperfect statement given of Dr. Maynard's inventions has extended this notice beyond just limits, but the innate modesty and delicate refinement of his nature would never allow him to obtrude his claims upon the public and the services he has rendered are too important to be suffered to pass into oblivion.

As one of the very few surviving contemporaries who was honored with his friendship, I offer this tribute to his memory. H. W. S. CLEVELAND.

